# Enhanced thermoelectric properties of AgSbTe_2_ obtained by controlling heterophases with Ce doping

**DOI:** 10.1038/s41598-017-04885-1

**Published:** 2017-07-03

**Authors:** Jae Ki Lee, Min-Wook Oh, Byungki Ryu, Ji Eun Lee, Bong-Seo Kim, Bok-Ki Min, Sung-Jae Joo, Hee-Woong Lee, Su-Dong Park

**Affiliations:** 1 Institute for Metallic Materials, Leibniz Institute for Solid State and Materials Research, Dresden, 01069 Germany; 2 Thermoelectric Conversion Research Center, Korea Electrotechnology Research Institute, Changwon, 51543 Korea; 3 Department of Advanced Materials Engineering, Hanbat National University, Daejeon, 34158 Korea

## Abstract

We report the enhanced thermoelectric properties of Ce-doped AgSbTe_2_ (AgSb_1−x_Ce_x_Te_2_) compounds. As the Ce contents increased, the proportion of heterophase Ag_2_Te in the AgSbTe_2_ gradually decreased, along with the size of the crystals. The electrical resistivity and Seebeck coefficient were dramatically affected by Ce doping and the lattice thermal conductivity was reduced. The presence of nanostructured Ag_2_Te heterophases resulted in a greatly enhanced dimensionless figure of merit, *ZT* of 1.5 at 673 K. These findings highlight the importance of the heterophase and doping control, which determines both electrical and thermal properties.

## Introduction

Thermoelectric (TE) power generators can be used to recycle waste heat from automobiles and incinerators. Although these devices are reliable and compact, the low energy conversion efficiency is a main drawback for TE power generation^1, 2^. The efficiency of TE power generators is limited by the temperature environment and TE figure of merit (ZT) of the materials used, as follows: *ZT* = *S* ^*2*^ *T*/*ρк*, where *S* is the Seebeck coefficient, *ρ* is the electrical resistivity, *κ* is the thermal conductivity, and *T* is temperature in Kelvin^3, 4^. Several efforts have been made to improve ZT.

Recently, the compound AgSbTe_2_ has been investigated as a potentially important component in high-performance bulk nanostructured TE materials, such as the LAST-m, ((PbTe)_m_(AgSbTe_2_))^5^ and TAGS-x ((GeTe)_x_(AgSbTe_2_)_100**−**x_)^6–9^. There is much interest in nanocomposite materials comprising heterophases which are embedded in the matrix^10–13^.

AgSbTe_2_ is also important by itself due to its good TE properties combined with relatively low thermal conductivity (0.6–0.7 W/mK)^14–16^. Wang *et al*. reported that AgSbTe_2_ has a high *ZT* = 1.59 at 673 K due to its extremely low thermal conductivity^16^. Also, some papers have reported good TE properties in the non-stoichiometric compositions formed by controlling the secondary phase, such as nanoscaled Ag_2_Te and Sb_2_Te_3_^17, 18^. Others have tried to not only reduce the lattice thermal conductivity but also adjust the carrier concentrations using dopants such as Na, Se, and Mn in AgSbTe_2_^19–21^; by suppressing the formation of impurity phases, they enhanced the TE properties. It is clear from the research mentioned that large-scale precipitates have a deleterious impact on TE properties, whereas the formation of nanoscale dispersion enhances these properties. Interfaces within a TE material have been shown to reduce the thermal conductivity without degrading the electrical conductivity^22, 23^.

In this work, we investigated the influence of Ce doping on the microstructure and TE properties of AgSb_1**−**x_Ce_x_Te_2_ as we varied the Ce concentration (x = 0–0.004). We found that the proportion of Ag_2_Te was significantly influenced by the Ce dopant, leading to improvements in the ZT value. To our knowledge, this is first observation of ZT enhancement arising from controlling the Ag_2_Te phase by doping. Du *et al*. reported on the effect of Ce on excess Te in AgSbTe_2.01_ compounds^24^. However, they did not sufficiently explain their results in terms of their impact on TE properties, particularly in relation to the morphology, or the effects of doping the compound with Ce. In comparison to their results, our results show considerably different transport properties. In practice, the carrier concentration and mobility were considerably different in our work. This difference may be attributable to a different fabrication process and the composition of the samples, which may result in different microstructures. A spark plasma sintering method was used in ref. 24., while a hot-pressing method was employed here to solidify samples, as well as the sintering temperature and pressure were also different, resulting in development of different morphologies. However, they did not report on the microstructure of their samples. In a related development, Marin *et al*.^25^. reported that the physical properties of single-crystalline samples of this material vary as a result of micro-fluctuations in the chemical composition. We demonstrate experimentally that doping AgSbTe_2_ systems with Ce is an effective approach for improving TE performance by reducing thermal conductivity and adjusting carrier concentrations; specifically, Ce doping enables control over the proportion of Ag_2_Te heterophases in the matrix AgSbTe_2_.

## Results and Discussion

**Figure 1:**
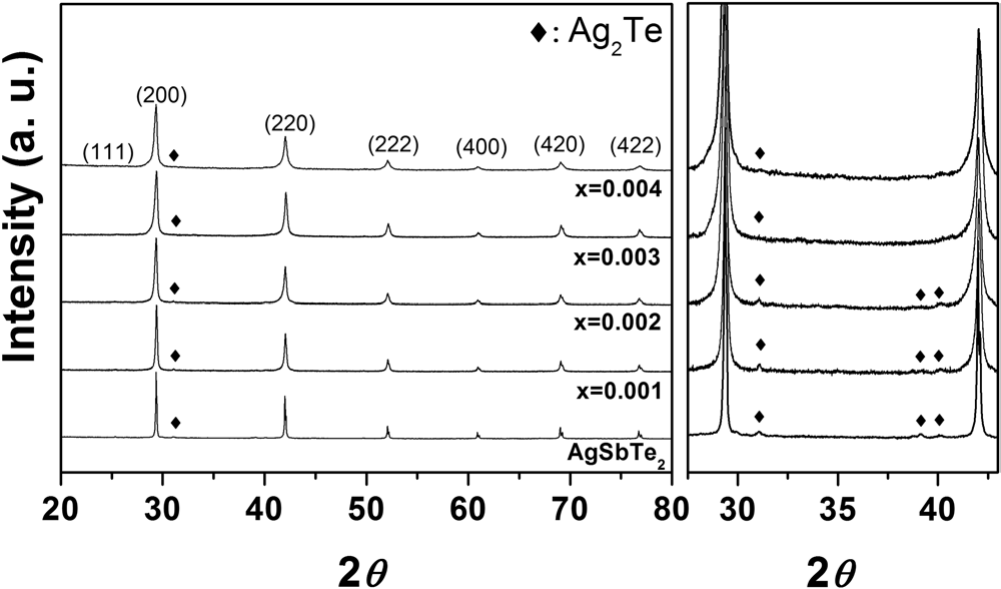
X-ray diffraction (XRD) patterns of Ce-doped AgSb_1−x_Ce_x_Te_2_ samples; the phase of the AgSbTe_2_ and the small amount of Ag_2_Te were detected in all samples; the right panel is the patterns focused on the region between 27.5 and 43 degrees.

The samples were obtained from sintering powders with hot-pressing method, in which the powders were obtained from melting elemental materials in quartz tubes with the nominal composition of AgSb_1**−**x_Ce_x_Te_2_ (0.001 ≤ x ≤ 0.004). Figure 1 shows the X-ray diffraction (XRD) pattern in Ce-doped AgSbTe_2_ compounds, obtained from the powder of the sintered samples. The phase of the AgSbTe_2_ and the small amount of Ag_2_Te were detected in all samples. The crystal structures of AgSbTe_2_ were identified as they caused disordered NaCl structures (space group, Fm$\bar{3}$m), in which the Ag and Sb were located at the Na site. A small amount of Ag_2_Te secondary phases were identified along with the main cubic phase. It should be noted that the exact crystal structure of AgSbTe_2_ is currently a subject of debate, so we do not know, for example, whether it is crystallized in *R* $\bar{3}$ *m*, *P4*/*mmm* and etc^26, 27^. We attempted to refine the AgSbTe_2_ structure based on the model of the disordered NaCl structure (*Fm* $\bar{3}$ *m*) and different models of ordered structures (*R* $\bar{3}$ *m*, *P4*/*mmm* and, *Fd* $\bar{3}$ *m*) using the Rietveld refinement method. Our Rietveld refinement analysis revealed that it was not easy to distinguish the crystal structure based on the statistical results from the refinement: the *R*_*wp*_ and *χ* ^2^ values are 7.85/2.08 in $Fm\bar{3}m$, 7.91/2.10 in $R\bar{3}m$, 7.71/2.45 in $P4/mmm$, and 7.76/2.05 in $Fd\bar{3}m$, respectively. Thus, we consider the space group of AgSbTe_2_ to maintain consistency with many other studies.

The cell parameters obtained from the refinement gradually decreased as the proportion of Ce increased. The small amount of Ce led to small changes: 0.6079 nm at x = 0; 0.6078 nm at x = 0.001; 0.6072 nm at x = 0.002; 0.6068 nm at x = 0.003; and 0.6066 nm at x = 0.004. This reduction was not attributed to the substitution of Ce for Sb or Ag, because the atomic or ionic radius of Ce is larger than that of Sb and Ag. Notably, it was found that the lattice parameters decreased with the amount of Ag_2_Te^18^. We speculate that the reduction in the lattice parameters is due to the reduction in the amount of Ag_2_Te. This speculation is supported by other observations detailed in the rest of this article.

Quantitative analysis of Ag_2_Te was carried out using the Rietveld refinement method. The results are shown in Table 1. The Ag_2_Te ratio decreased from 9.5% to 2.0%; the peaks also broadened with Ce doping. The peak broadening was analyzed using the Williamson–Hall equation:

1 $$\frac{\beta \,\cos \,\theta }{\lambda }=\frac{1}{d}+4\varepsilon \frac{\sin \,\theta }{\lambda }$$

**Table 1:** Rietveld refinement results of the Ce-doped AgSb_1−x_Ce_x_Te_2_ samples.

Sample	Detected Ce contents (ICP)	*R*_*exp*_	*R*_*wp*_	*χ* ^2^	*Ag*_*2*_ *Te wt*%	*Cation sites occupancy*		
						*Ag*	*Sb*	*Ag*/*Sb*
AgSbTe_2_	Non	3.78	7.85	2.08	9.5%	0.754	1.074	0.702
x = 0.001	Non	7.07	8.23	1.16	6.2%	0.772	1.078	0.716
x = 0.002	x = 0.0037	4.81	8.00	1.66	6.0%	0.784	1.066	0.735
x = 0.003	x = 0.0056	4.72	9.79	2.07	4.2%	0.97	1.136	0.854
x = 0.004	x = 0.0111	3.89	9.66	2.48	2.0%	0.974	1.136	0.857

where *β* is the integral breadth of the diffraction peak, *λ* is the X-ray wavelength, *d* is the average grain size, *ѳ* is the Bragg diffraction angle, and *ε* is the microscopic strain^28^. The crystal size decreased as the amount of Ce increased: 71.8 nm at x = 0; 55.9 nm at x = 0.001; 47.3 nm at x = 0.002; 35.4 nm at x = 0.003; and 34.5 nm at x = 0.004.

**Figure 2:**
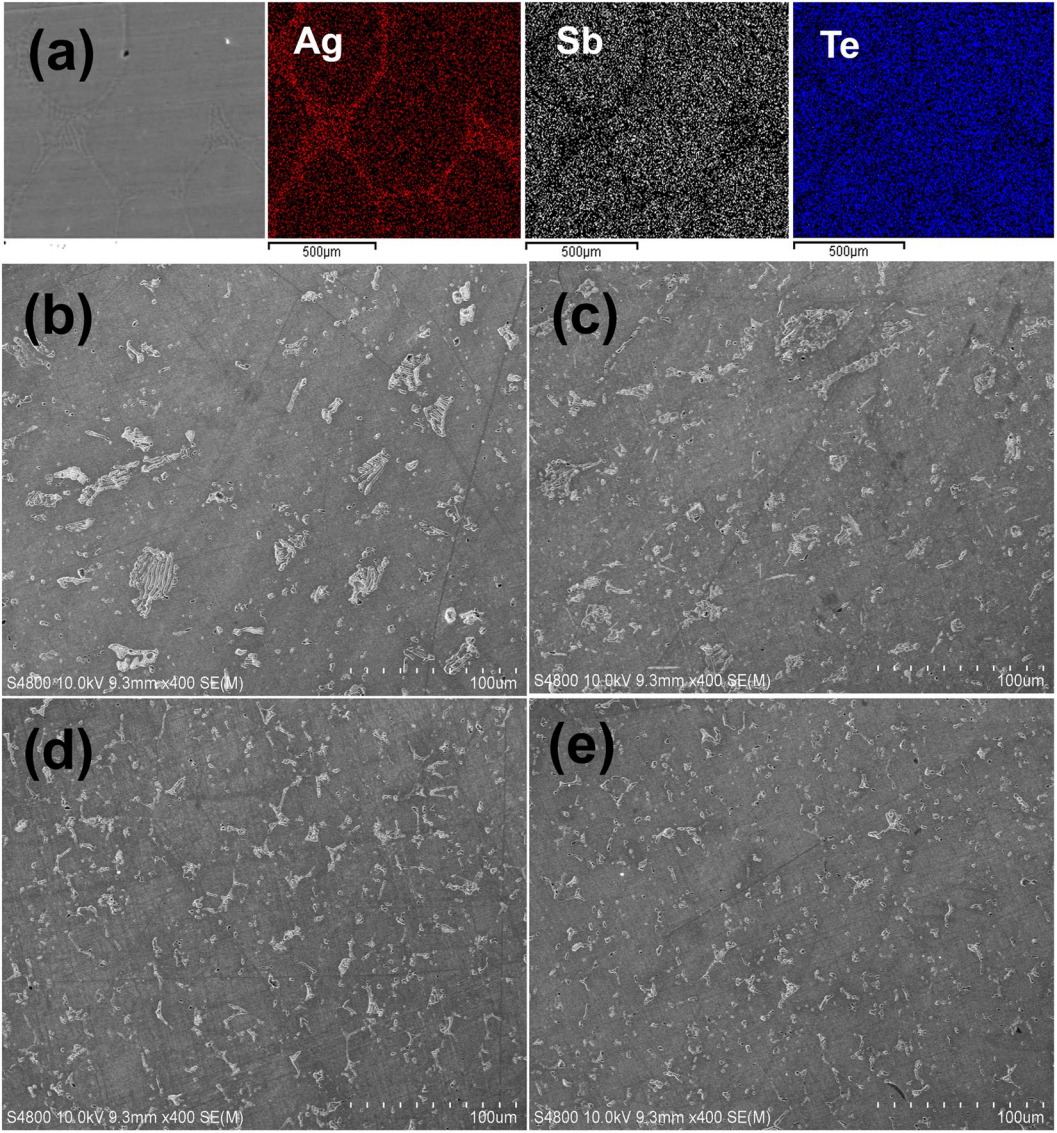
(**a**) SEM images and the elemental distribution of AgSbTe_2_ ingots quenched after melting; the region of grain boundaries was identified as Ag_2_Te precipitates, (**b**–**e**) SEM images of the hot-pressed samples; the light and dark regions are Ag_2_Te precipitates and AgSbTe_2_ matrix, respectively; the precipitates crushed from melted ingots remained as isolated precipitates in the sintered samples, (**b**) AgSbTe_2_, (**c**) x = 0.002, (**d**) x = 0.003, and (**e**) x = 0.004 samples in AgSb_1−x_Ce_x_Te_2_.

To determine the microstructure of the compound, SEM images were obtained (Fig. 2). The continuously segregated precipitates of Ag_2_Te in the melted ingot at the grain boundaries are clearly shown in Fig. 2(a), in which the SEM and elemental mapping images of the ingots obtained after the melting–quenching cycle are shown. It is clear that the Ag_2_Te phase formed after the solidification of AgSbTe_2_. The segregated precipitates were crushed during pulverization of the ingot and remained as isolated precipitates in the sintered samples, as shown in Fig. 2(b). The light and dark regions were Ag_2_Te precipitates and AgSbTe_2_ matrix, respectively. Nano-sized precipitates were not observed in the images. Additionally, the amount of Ag_2_Te precipitate decreased as the amount of Ce increased, as shown in Fig. 2(c–e). The size of each precipitate was also reduced, in agreement with our XRD analysis.

**Figure 3:**
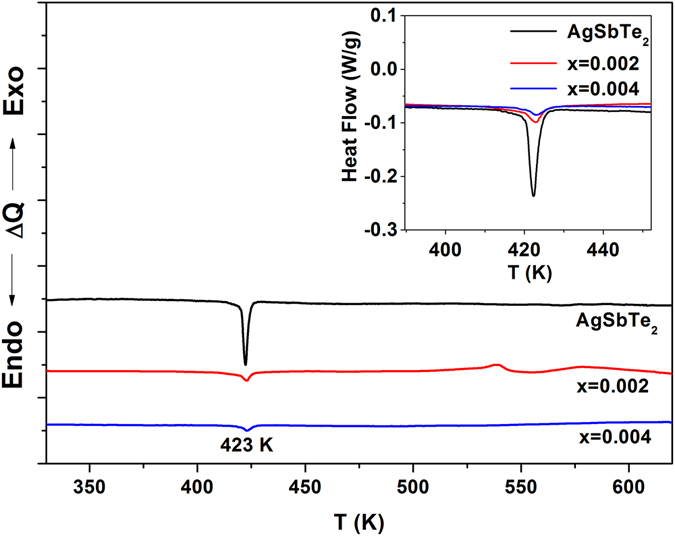
Differential scanning calorimetry (DSC) curves for the Ce-doped AgSb_1−x_Ce_x_Te_2_ samples; the peak at 423 K corresponds to the phase transition peaks from *α*-Ag_2_Te to *β*-Ag_2_Te. The intensity decreased with Ce doping, which is related with the decreased Ag_2_Te ratio. (The sample mass is 50 mg.)

The reduced amount of Ag_2_Te was also confirmed by differential scanning calorimetry (DSC) measurements. The endothermic peaks at 423 K can be observed in the DSC curves shown in Fig. 3. The peak at 423 K corresponds to the phase transition peaks from *α*-Ag_2_Te to *β*-Ag_2_Te^29^. The intensity of the peak at 423 K is distinctly lower for the x = 0.002 and the x = 0.004 samples. This is due to the decreased Ag_2_Te ratio. We confirmed the reduction in the proportion of Ag_2_Te in Ce-doped AgSbTe_2_ using a variety of analyses. At this stage, one may argue that the reduction in the amount of Sb in the studied samples may lead to the reduction of Ag_2_Te. From the phase diagram between Ag_2_Te and Sb_2_Te3^30, 31^, we can easily see that the amount of Ag_2_Te is lower in Sb-rich AgSbTe_2_. Thus, the reduction in the proportion of Ag_2_Te can be attributed to the addition of Ce. The reduction in Ag_2_Te with such a small amount of Ce may be due to the following: It is noteworthy that the rock salt-structured compound exists in a range of stable compositions between Ag_0.76_Sb_1.16_Te_2.08_, and Ag_0.88_Sb_1.12_Te_2_ and the cations are disordered in Sb-rich compositions^32^. First-principles calculations also showed that Ag_Sb_ and Sb_Ag_ antisite defects can be favorably formed in AgSbTe_2_^33^. The Sb-rich composition of AgSbTe_2_ is attributed to the formation of Sb_Ag_. The formation of antisite defects is dependent on the difference in the electronegativity between two elements; the values for Ag and Sb are 1.93 and 2.05, respectively^34, 35^. If the averaged difference of the electronegativity of the cations changes, the behavior of the antisite defect formation will also change^35, 36^. It is known that the electronegativity of Ce is 1.12. Hence, the averaged difference would change as Ce is added, resulting in Ag occupying more cation sites in AgSbTe_2_ and a reduction in the proportion of Ag_2_Te. It is clearly shown that ratio of cation sites occupancies of Ag to Sb in AgSbTe_2_ increased with Ce doping as indicated in Table 1.

**Figure 4:**
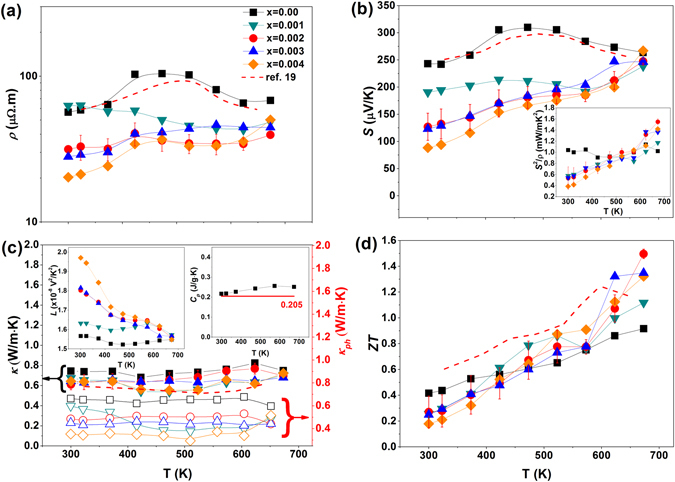
Effect of temperature on the thermoelectric (TE) properties of Ce-doped AgSb_1−x_Ce_x_Te_2_: (**a**) electrical resistivity, (**b**) Seebeck coefficient; the inset shows the power factors, (**c**) total thermal conductivity (solid shapes) and lattice thermal conductivity (open shapes); the left and right insets show the Lorenz number and the heat capacity, respectively. (The red line of 0.205 J/g∙K in the right inset in (**c**) represents the literature value from ref. 14.), and (**d**) the dimensionless figure of merit (*ZT*). The red dashed lines in all four panels are the corresponding data from ref. 19., while that in (**c**) is the total thermal conductivity from ref. 19.

Figure 4(a) and (b) show the effect of temperature on the electrical resistivity (*ρ*) and Seebeck coefficient (*S*) of Ce-doped AgSbTe_2_ compounds. It is well known that the electrical resistivity depends on the carrier concentration and mobility according to *ρ* = *1*/*enμ*, where *μ* and *e* are the mobility and carrier charge, respectively. Table 2 shows the carrier concentration and the mobility of Ce-doped AgSbTe_2_ compounds at room temperature. The positive Seebeck coefficient and Hall coefficient indicate that the major carriers are holes. The carrier concentration increased with the amount of Ce added, whereas the mobility of the Ce-doped compound was lower than that of the undoped compound. Thus, the reduced electrical resistivity of the Ce-doped compound, except for x = 0.001, was mainly caused by changes in the carrier concentration. The reduction in the Seebeck coefficient as Ce is added can also be attributed to the carrier concentration, because S depends on *S* ~ *p* ^**−**2/3^ in highly degenerate semiconductors^3^. The reduced mobility is thought to be due to the finer crystallite size in the Ce-doped AgSbTe_2_. Ag_2_Te acts as a minor carrier in the p-type AgSbTe_2_ matrix, so by decreasing the proportion of Ag_2_Te, the carrier concentration can increase^17, 37^.

**Table 2:** Carrier concentration and the mobility of Ce-doped AgSb_1−x_Ce_x_Te_2_ samples.

Sample	*h* (/cm^3^)	*μ* (cm^2^/Vs)
AgSbTe_2_	5.55 × 10^18^	190
x = 0.001	6.59 × 10^18^	163
x = 0.002	1.32 × 10^19^	143
x = 0.003	1.27 × 10^19^	175
x = 0.004	1.57 × 10^19^	157

The broad bump in the temperature dependence of the *ρ* and *S* from AgSbTe_2_ is interesting. It is known that the abrupt changes at ~423 K in *ρ* and *S* are related to a phase transition from *α*-Ag_2_Te to *β*-Ag_2_Te^18^. This bump was not clearly observed in the Ce-doped compound, which may be due to the reduction in the proportion of Ag_2_Te. The values of the electrical resistivity at high temperatures were smaller for the Ce-doped compound. This is also thought to be due to the reduction in Ag_2_Te, which can be a source of scattering.

The temperature dependence of the power factor (*S* ^*2*^ */ρ)* is shown in the inset of Fig. 4(b). The maximum value of 1.55 mW/m·K^2^ at 673 K was achieved in the x = 0.002 sample. The enlarged power factor at elevated temperatures for the Ce-doped compound was mainly due to the reduced electrical resistivity.

In Fig. 4(c), the solid shapes represent the temperature dependence of the total thermal conductivity (*κ*), while open/empty shapes are the lattice thermal conductivity (*κ*_*ph*_) of the Ce-doped AgSbTe_2_ compounds. The lattice thermal conductivity can be evaluated by subtracting the electronic contribution (*κ*_*e*_) from the total thermal conductivity (*κ*), *κ*_*ph*_ = *κ* − *κ*_*e*_. *κ*_*e*_ can be estimated using the Wiedemann–Franz law, *κ*_*e*_ = *LT/ρ*, in which *L* is the Lorenz number. The value of *L* is largely dependent on the position of the Fermi level and the scattering mechanism. It can be calculated using:

2 $$L={(\frac{k}{e})}^{2}\frac{(1+\lambda )(3+\lambda ){F}_{\lambda }(\eta ){F}_{(2+\lambda )}(\eta )-{(2+\lambda )}^{2}{F}_{(1+\lambda )}{(\eta )}^{2}}{{(1+\lambda )}^{2}{F}_{\lambda }{(\eta )}^{2}}$$

where *λ* is the scattering factor (*λ* = 0 for acoustic phonon scattering and *λ* = 2 for ionized impurity scattering) and ${F}_{r}(\eta )={\int }_{0}^{\infty }{\xi }^{r}{f}_{0}(\eta )d\xi$, where *f*_0_ is the Fermi distribution, _*ξ*_ i s the reduced energy of the carriers, and *η* is the reduced Fermi energy^35, 38^. Assuming acoustic phonon scattering and determining the value of *η* from the measured Seebeck coefficient according to Eq. (3), we were able to calculate the value of *L*, which is shown in the inset of Fig. 4(c):

3 $$S=(\frac{k}{e})\{\frac{(2+\lambda ){F}_{1+\lambda }}{(1+\lambda ){F}_{\lambda }}-\eta \}.$$

All Ce-doped samples had lower total thermal conductivity and lower lattice thermal conductivity than the ternary compound. The lattice thermal conductivity for the x = 0.002 sample was ~0.44 W/m∙K at room temperature and ~0.43 W/m∙K at 673 K, which is ~30% lower than the lattice thermal conductivity of the ternary compound (*κ*_*ph*_ = 0.66 and 0.6 W/m∙K at room temperature and 673 K, respectively). The total thermal conductivity was in the range of 0.6–0.8 W/m∙K, similar to that observed in a previous report^14^. In the inset of Fig. 4(c), the measured specific heat capacity (*Cp*) of the AgSbTe_2_ samples was used to calculate the total thermal conductivity. The values are 0.217 J/g∙K at room temperature and 0.251 J/g∙K at 673 K; these values are significantly higher than the literature value (*Cp = *0.205 J/g∙K) of AgSbTe_2_^14^.

**Figure 5:**
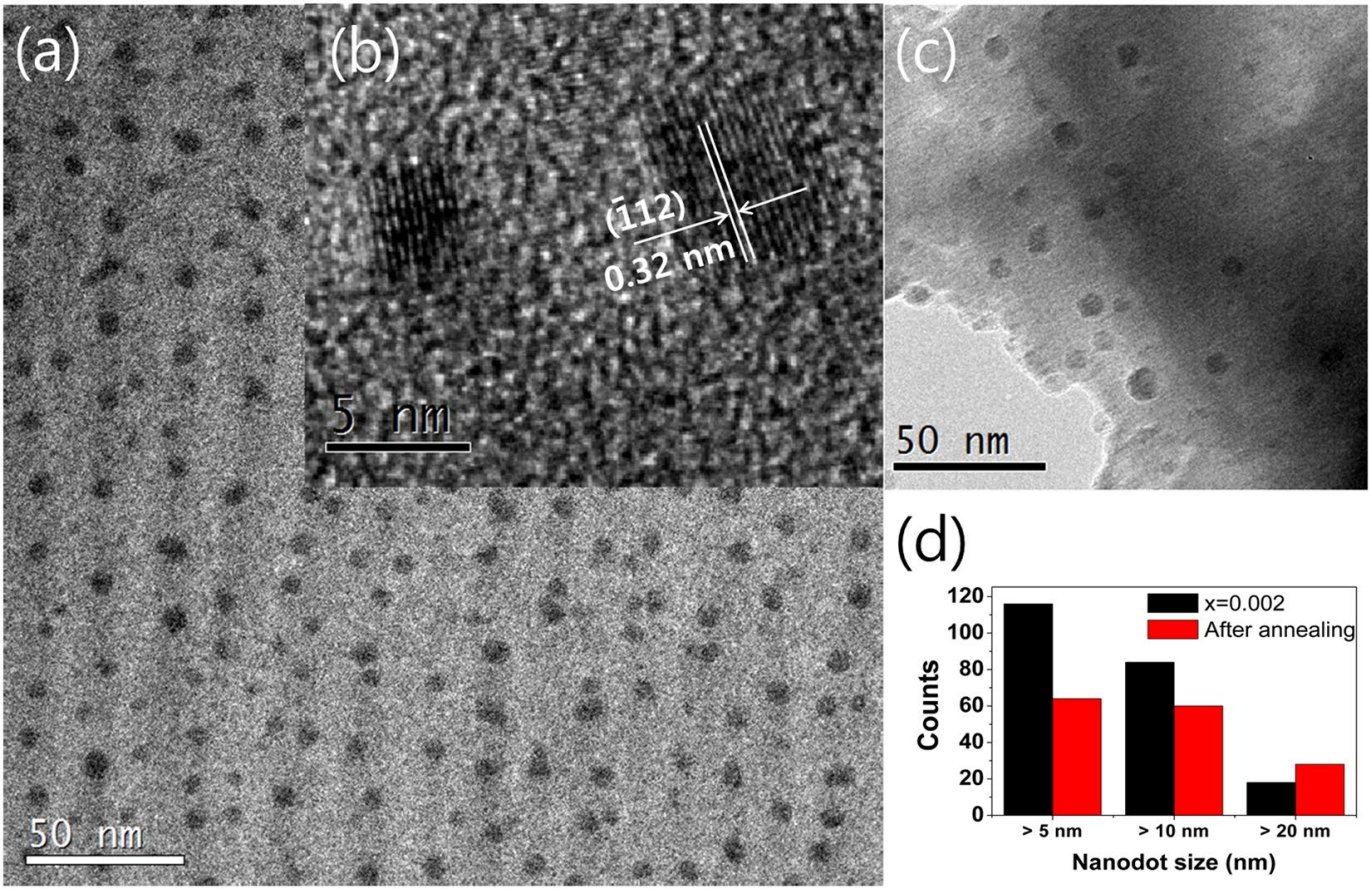
(**a**) Transmission electron microscopy (TEM) images of the x = 0.002 sample in AgSb_1−x_Ce_x_Te_2_, (**b**) magnified images of Ag_2_Te nanostructures, (**c**) TEM images after annealing at 673 K for 24 h, and (**d**) histogram of nanodots with respect to size.

The reduction in *κ* as Ce was added was mainly due to the reduction in *κ*_*ph*_. To investigate the origin of the reduced *κ*_*ph*_, we observed the nanoscale microstructures of the samples using TEM. The images obtained are shown in Fig. 5. The nanostructures with a size of about 10 nm were identified as Ag_2_Te, which is expected from the *d*-spacing (0.32 nm) of $(\bar{1}12)$ ^32^. This finding contrasts with the image of AgSbTe_2_ matrix. Only the Ag_2_Te monoclinic phase has the same *d*-spacing size in the possible phases like Ag_2_Te, Sb_2_Te_3_, and AgSbTe_2_ in this system. Also, if the nanodots are the Sb_2_Te_3_ phase, it will be shown brightly compared to AgSbTe_2_ matrix in the bright field image of TEM^39^. The difference in the size of the nanostructures was insignificant after annealing at 673 K for 24 h, as shown in Fig. 5(c) and (d). It is well known that the nanostructures are major phonon scattering centers and lead to a reduction in the level of lattice thermal conductivity. For example, 80% of phonons in PbTe have a mean free path below 100 nm and are effectively scattered by nanostructures^10^. Thus, we think that the reduced *κ*_*ph*_ of the Ce-doped samples was mainly caused by the existence of nanostructures, as nanostructured Ag_2_Te was not observed in the ternary compound. Furthermore, the finer crystallite sizes in the Ce-doped compound also contribute marginally to the reduction in *κ*_*ph*_.

**Figure 6:**
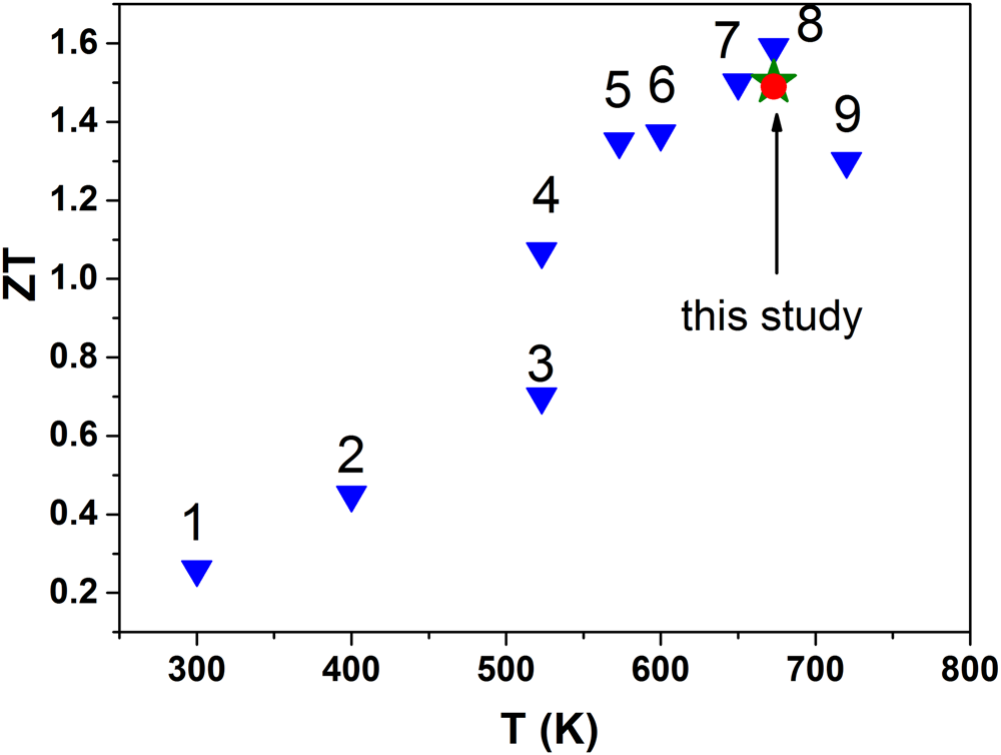
Comparison of the ZT values:Data 1~9 are from ref. 13, 40﻿, 41, 42, 20, 17, 19, 16 and 43, respectively.

Figure 4(d) shows the effect of temperature on ZT. The value of *ZT = *0.92 at 673 K for the undoped sample is lower than the value reported in the literature (ZT = ~1.2)^19, 20, 24^. The measured value of *κ* was larger than previously reported, although the values of *S* and *ρ* were similar. We speculate that the difference is due to the use of a much higher value of *Cp*, although we do not know what value was used in previous studies. The maximum value of ZT was 1.5 at 673 K. This was obtained using the x = 0.002 sample. This ZT value is ~39% higher than that of the undoped sample. The thermal stability of the TE performance was analyzed after annealing at 673 K for 24 h. The ZT value was almost sustained at 1.49 at 673 K. The reductions in *κ*_*ph*_ and *ρ* contributed significantly to the enhancement in ZT. The obtained ZT is as high as the highest reported values, as shown in Fig. 6, even though the report had shown very low density, from which low *κ*_*ph*_ was obtained, and no experimental value of the specific heat capacity^16, 19^. It is noteworthy that the enhanced ZT value was achieved with high density (over 97%), which means AgSbTe_2_ has a range of possible applications.

### Summary

We developed Ce-doped AgSbTe_2_ compounds that exhibit enhanced TE performance. The addition of Ce influenced the formation of the heterophase Ag_2_Te in the AgSbTe_2_ matrix. We used a variety of methods to confirm that the proportion of Ag_2_Te was reduced as Ce was added. The change in the proportion of Ag_2_Te with the addition of Ce was thought to be responsible for the variation in the electrical and thermal properties. We obtained reduced *ρ* and *κ* values. This resulted in a particularly high *ZT* = 1.5 at 673 K within highly densified samples. Hence, the Ce-doped AgSbTe_2_ has promising practical applications. It is remarkable that both electrical and thermal properties can be optimized by controlling the proportion of heterophases, so that high *ZT* values can be achieved.

## Method

### Materials and characterization

The Ce-doped AgSbTe_2_ compounds were fabricated by a conventional melting, quenching, and hot-pressing method^6, 17, 18^. Each element (Ce, 99.9%; Ag, 99.999%, Sb, 99.999% and Te, 99.999% in purity) was weighed in a glove box with the nominal composition of AgSb_1−x_Ce_x_Te_2_ (0.001 ≤ x ≤ 0.004). The admixture of elements was directly loaded into quartz tubes and was sealed under Ar atmosphere. The sealed ampoules were heated to 1233 K for 10 h in a rocking furnace and quenched in ice water. The solidified ingots were crushed, sieved to particles of <45 μm, and sintered by hot pressing in Ar for 20 min at 683 K with a pressure of 100 MPa. The samples of 12.7 mm in diameter and about 15 mm in height were cut by a diamond wire saw and polished. The real compositions of the sintered samples were examined using inductively coupled plasma (ICP).

The phases of the Ce-doped AgSbTe_2_ compounds were analyzed with an X-ray diffractometer (x’pert pro, Analytical) using Cu-Kα radiation (λ = 0.15406 nm). The microstructure and local composition of the ingot were investigated by field- emission scanning electron microscopy (FE-SEM, S-4800, Hitachi) with energy dispersive X-ray spectroscopy (EDS) and field-emission transmission electron microscopy (FE-TEM, Tecnai G^2^ F30 S-twin, FEI) using an electron microprobe. The electrical properties were analyzed in terms of the Seebeck coefficient and resistivity measurement system (ZEM-3, ULVAC-RIKO). The thermal diffusivity was measured using the laser flash method (LFA-457, NETZSCH). The heat capacity was obtained using a differential scanning calorimeter (DSC 404 C, NETZSCH). The method of Archimedes was applied to measure the density of samples. Thermal conductivity was calculated from the density (*d*), heat capacity (*Cp*), and thermal diffusivity (*a*), using the equation: *κ* = *a ∙ Cp ∙ d*. The Hall effect measurement, conducted with a magnet of 0.55 T, provided information on the carrier concentration and mobility.

## References

[1] Ma J (2013). Glass-like phonon scattering from a spontaneous nanostructure in AgSbTe_2_. Nature nanotechnology.

[2] DiSalvo FJ (1999). Thermoelectric cooling and power generation. Science.

[3] Son JS (2012). n-type nanostructured thermoelectric materials prepared from chemically synthesized ultrathin Bi_2_Te_3_ nanoplates. Nano Lett..

[4] Dresselhaus MS (2007). New directions for low-dimensional thermoelectric materials. Adv. Mater..

[5] Hsu KF (2004). Cubic AgPb_m_SbTe_2+m_: Bulk thermoelectric materials with high figure of merit. Science.

[6] Yang SH (2008). Nanostructures in high-performance (GeTe)_x_(AgSbTe_2_)_100−x_ thermoelectric materials. Nanotechnology.

[7] Cook BA, Kramer MJ, Wei X, Harringa JL, Levin EM (2007). Nature of the cubic to rhombohedral structural transformation in (AgSbTe_2_)_15_(GeTe)_85_ thermoelectric material. J. Appl. Phys..

[8] Christakudis GC, Plachkova SK, Shelimova LE, Avilov ES (1991). Thermoelectric figure of merit of some compositions in the system (GeTe)_1−x_[(Ag_2_Te)_1−y_(Sb_2_Te_3_)_y_]_x_. Phys. Status Solidi. A.

[9] Rowe, D. M. CRC Handbook of Thermoelectrics, (ed Rowe, D. M.) 267–275. (CRC Press, 1995).

[10] Biswas K (2012). High-performance bulk thermoelectrics with all-scale hierarchical architectures. Nature.

[11] Fan S (2010). p-type Bi_0.4_Sb_1.6_Te_3_ nanocomposites with enhanced figure of merit. Appl. Phys. Lett..

[12] Cao YQ, Zhao XB, Zhu TJ, Zhang XB, Tu JP (2008). Syntheses and thermoelectric properties of Bi_2_Te_3_/Sb_2_Te_3_ bulk nanocomposites with laminated nanostructure. Appl. Phys. Lett..

[13] Sharma PA, Sugar JD, Medlin DL (2010). Influence of nanostructuring and heterogeneous nucleation on the thermoelectric figure of merit in AgSbTe_2_. J. Appl. Phys..

[14] Morelli DT, Jovovic V, Heremans JP (2008). Intrinsically minimal thermal conductivity in cubic I-V-VI_2_ semiconductors. Phys. Rev. Lett..

[15] Wojciechowski K, Tobola J, Schmidt M, Zybala R (2008). Crystal structure, electronic and transport properties of AgSbSe_2_ and AgSbTe_2_. J. Phys. Chem. Solids.

[16] Wang H, Li J-F, Zou M, Sui T (2008). Synthesis and transport property of AgSbTe_2_ as a promising thermoelectric compound. Appl. Phys. Lett..

[17] Zhang SN, Zhu TJ, Yang SH, Yu C, Zhao XB (2010). Phase compositions, nanoscale microstructures and thermoelectric properties in Ag_2−y_Sb_y_Te_1+y_ alloys with precipitated Sb_2_Te_3_ plates. Acta Mater..

[18] Zhang SN, Zhu TJ, Yang SH, Yu C, Zhao XB (2010). Improved thermoelectric properties of AgSbTe_2_ based compounds with nanoscale Ag_2_Te *in situ* precipitates. J. Alloy. Compd..

[19] Du B, Li H, Tang XF (2011). Enhanced thermoelectric performance in Na-doped p-type nonstoichiometric AgSbTe_2_ compound. J. Alloy. Compd..

[20] Du B, Li H, Xu J, Tang X, Uher C (2010). Enhanced figure-of-merit in Se-doped p-type AgSbTe_2_ thermoelectric compound. Chem. Mater..

[21] Zhang H (2012). Synthesis and thermoelectric properties of Mn-doped AgSbTe_2_ compounds. Chin. Phys. B.

[22] Poudel B (2008). High-thermoelectric performance of nanostructured bismuth antimony telluride bulk alloys. Science.

[23] Medlin DL, Snyder GJ (2009). Interfaces in bulk thermoelectric materials: A review for current opinion in colloid and interface science. Curr. Opin. Colloid Interface Sci..

[24] Du B, Li H, Tang X (2014). Effect of Ce substitution for Sb on the thermoelectric properties of AgSbTe_2_ compound. J. Electron. Mater..

[25] Ayral-Marin RM, Brun G, Maurin M, Tedenac JC (1990). Contribution to the study of AgSbTe_2_. Eur. J. Solid State Inorg. Chem..

[26] Quarez E (2005). Nanostructuring, compositional fluctuations, and atomic ordering in the thermoelectric materials AgPb_m_SbTe_2+m_. The myth of solid solution. J. Am. Chem. Soc..

[27] Ko YH (2014). Structural studies of AgSbTe_2_ under pressure: Experimental and theoretical analyses. Curr. Appl. Phys..

[28] Williamson GK, Hall WH (1953). X-ray line broadening from filed aluminium and wolfram. Acta. Metall.

[29] Ragimov SS, Aliev SA (2007). α-β phase transition of Ag_2_Te in the AgSbTe_2_ alloy of the Ag-Sb-Te system. Inorg. Mat..

[30] Marin RM, Brun G, Tedenac JC (1985). Phase equilibria in the Sb_2_Te_3_-Ag_2_Te system. J. Mater. Sci..

[31] Majer RG (1963). Zur Kenntnis des Systems PbTe–AgSbTe_2_. Zeitschrift Fur Metallkunde.

[32] Sugar JD, Medlin DL (2009). Precipitation of Ag_2_Te in the thermoelectric material AgSbTe_2_. J. Alloy Compd..

[33] Barabash SV, Ozolins V, Wolverton C (2008). First-principles theory of the coherency strain, defect energetics, and solvus boundaries in the PbTe-AgSbTe_2_ system. Phys. Rev. B.

[34] Allred AL (1961). Electronegativity values from thermochemical data. J. Inorg. Nucl. Chem..

[35] Son JH (2013). Effect of ball milling time on the thermoelectric properties of p-type (Bi,Sb)_2_Te_3_. J. Alloy Compd..

[36] Oh MW (2014). Antisite defects in n-type Bi_2_(Te,Se)_3_: Experimental and theoretical studies. J. Appl. Phys..

[37] Xu J (2010). High thermoelectric figure of merit and nanostructuring in bulk AgSbTe_2_. J. Mater. Chem..

[38] May AF, Fleurial JP, Snyder GJ (2008). Thermoelectric performance of lanthanum telluride produced via mechanical alloying. Phys. Rev. B.

[39] Medlin DL, Sugar JD (2010). Interfacial defect structure at Sb_2_Te_3_ precipitates in the thermoelectric compound AgSbTe_2_. Scripta Materialia.

[40] Jovovic V, Heremans JP (2008). Measurements of the energy band gap and valence band structure of AgSbTe_2_. Phys. Rev. B.

[41] Wojciechowski KT, Schmidt M (2009). Structural and thermoelectric properties of AgSbTe_2_-AgSbSe_2_ pseudobinary system. Phys. Rev. B.

[42] Su T (2009). Enhanced thermoelectric performance of AgSbTe2 synthesized by high pressure and high temperature. Appl. Phys. Lett..

[43] Wood C (1988). Materials for thermoelectric energy conversion. Rep. Prog. Phys..

